# Hybrid Histidine Kinase WelA of *Sphingomonas* sp. WG Contributes to WL Gum Biosynthesis and Motility

**DOI:** 10.3389/fmicb.2022.792315

**Published:** 2022-03-01

**Authors:** Hui Li, Mengqi Chen, Zaimei Zhang, Benchao Li, Jianlin Liu, Han Xue, Sixue Ji, Zhongrui Guo, Jiqian Wang, Hu Zhu

**Affiliations:** ^1^State Key Laboratory of Heavy Oil Processing and Centre for Bioengineering and Biotechnology, China University of Petroleum (East China), Qingdao, China; ^2^Engineering Research Center of Industrial Biocatalysis, Fujian Province Universities, College of Chemistry and Materials Science, Fujian Normal University, Fuzhou, China

**Keywords:** *Sphingomonas* sp. WG, WelA, regulatory pathway, WL gum, c-di-GMP, flagellar assembly

## Abstract

*Sphingomonas* sp. WG produced WL gum with commercial utility potential in many industries. A hybrid sensor histidine kinase/response regulator WelA was identified to regulate the WL gum biosynthesis, and its function was evaluated by gene deletion strategy. The WL gum production and broth viscosity of mutant Δ*welA* was only 44% and 0.6% of wild type strain at 72 h. The transcriptomic analysis of differentially expressed genes showed that WelA was mapped to CckA; ChpT, and CtrA in the CckA-ChpT-CtrA pathway was up-regulated. One phosphodiesterase was up-regulated by CtrA, and the intracellular c-di-GMP was decreased. Most genes involved in WL gum biosynthesis pathway was not significantly changed in Δ*welA* except the up-regulated *atrB* and *atrD* and the down-regulated *pmm*. Furthermore, the up-regulated regulators *ctrA, flaEY*, *flbD*, and *flaF* may participate in the regulation of flagellar biogenesis and influenced motility. These results suggested that CckA-ChpT-CtrA pathway and c-di-GMP were involved in WL gum biosynthesis regulation. This work provides useful information on the understanding of molecular mechanisms underlying WL gum biosynthesis regulation.

## Introduction

Exopolysaccharides (EPSs) secreted by many bacterial strains are ubiquitous and have a wide range of industrial applications, including emulsification, thickening, and pharmaceutical applications (Kumar et al., 2007; Freitas et al., 2011; Schmid et al., 2015). Several EPSs such as xanthan, gellan, curdlan, and succinoglucan have been commercially produced and applied in recent years (Lelchat et al., 2015). Sphingans such as gellan, S-88, welan, and diutan are EPSs produced by the genus *Sphingomonas* sp. They have a similar repeating tetrasaccharide backbone structure as glucose-glucuronic acid-glucose-rhamnose or mannose (Fialho et al., 2008). It has been proved that the known sphingans with special side chains have unique rheological characteristics and commercial applications in petroleum, food, concrete, and pharmaceutical industries as gelling, emulsifiers, stabilizers, and viscosifiers agents (Fialho et al., 2008). WL gum was one novel sphingan produced by the strain *Sphingomonas* sp. WG with high viscosity, pseudoplastic behavior, good resistance to high temperature and high concentration of salts, stability to a broad range of pH (1–14). As a result, it can be used in food, concrete, enhanced oil recovery, and many other industries (Li et al., 2016b).

Due to these valuable properties, studies were performed to improve the WL gum production. For example, the medium composition and fermentation parameters optimization strategy has been used, and the WL gum production reached 39.95 g/L, 2.37-fold of the initial production (16.82 g/L) (Li et al., 2018). However, the normal optimization of the fermentation process is restricted by the physiological limitation of strains. Therefore, metabolic engineering with increased sphingans production or volumetric productivity attracted more attention. A further understanding for the fundamental biosynthesis process and regulation mechanism is critical for genetic and metabolic engineering approaches. In our previous work, the genome of *Sphingomonas* sp. WG was sequenced and annotated (Li et al., 2016a), and the *wel* cluster for WL gum biosynthesis was predicted. Similar to the previously reported sphingans [gellan gum (Harding et al., 2004), S88 (Yamazaki et al., 1996), diutan gum (Coleman et al., 2008), and welan gum (Schmid et al., 2014)], the biosynthesis of WL gum might follow a Wzx/Wzy-dependent pathway and three sequential steps involved: (i) intracellular synthesis of nucleotide-sugar precursors catalyzed by PGM, UGP, UGD, etc.; (ii) assembly of the tetrasaccharide repeating units by different glycosyltransferases; (iii) polymerization of the assembled repeating units to form longer chains and export of the polysaccharide in the general Wzx/Wzy-dependent pathway. Our previous work has characterized the function of some enzymes, including PGM, UGP, and WelK (Li et al., 2019, 2021). The over-expression of a single gene or gene clusters for other sphingans was also employed to enhance the carbon flux toward the final polymer. However, it had a negative effect in many cases (Thorne et al., 2000; Sa-Correia et al., 2002). Therefore, the elucidation of the regulation mechanism of sphingan biosynthesis will allow the understanding of the bottlenecks of the sphingan biosynthesis pathway and provide the target for metabolic engineering.

The putative protein GelA, which is homologous to hybrid sensor kinase and response regulatory protein, was supposed to be involved in the gellan biosynthesis regulation because the mutants were non-mucoid on solid YM medium (Harding et al., 2004; Fialho et al., 2008). The over-expression of *gelA* in *Sphingomonas elodea* resulted in enhanced gellan biosynthesis, which might be related to the increased transportation of sugar units through comparative two-dimensional gel electrophoresis analysis (Lee et al., 2017). However, the signaling pathway of GelA in the regulation of gellan gum biosynthesis was still unclear. *welA*, a homologous version of *gelA* (78% identity), is annotated in the draft genome of *Sphingomonas* sp. WG and proposed to encode the regulator for WL gum biosynthesis. Therefore, in this work, *welA* was knocked out from the genome of *Sphingomonas* sp. WG and its effects on WL gum production were investigated. Furthermore, the transcriptomic analysis was performed between the wild strain and the mutant strain with the aim of better understanding the metabolic pathways such as motility and WL gum biosynthesis regulated by WelA. The results obtained in this manuscript provided useful information on molecular mechanisms underlying the regulation of WL gum biosynthesis.

## Materials and Methods

### Strains, Plasmids, Culture Conditions, and Chemicals

The strain *Sphingomonas* sp. WG (CCTCC No. M2013161) was maintained on Luria-Bertani (LB) agar slants at 4°C and was activated in seed medium (10 g/L glucose, 1 g/L yeast extract (YE), 5 g/L tryptone, 2 g/L KH_2_PO_4_, and 0.1 g/L MgSO_4_) at 28°C. The activated strain was sequentially transferred to a 250 mL flask containing 50 mL fermentation medium (67 g/L glucose, 3.4 g/L YE, 3 g/L K_2_HPO_4_, 0.1 g/L MgSO_4_, pH 7.0; Zhou et al., 2017), and incubated at 32°C, 150 rpm for 72 h to produce WL gum. To amplify the recombinant plasmid DNA, *Escherichia coli* DH5α was chosen as the host strain, and related recombinant *E. coli* DH5α strains were the donor strains in bacterial conjugation. *E.* coli DH5α was cultivated at 37°C in LB medium supplemented with tetracycline or gentamicin when necessary. The plasmid pJQ200SK containing the *sacB* gene as the counter-selectable marker (Pelicic et al., 1996) was applied to construct the *welA* deletion strain △*welA*. The plasmid pBBR1MCS-3 (Kovach et al., 1995) was used to introduce *welA* gene into the △*welA* to construct the complemented strain △*welA*/pBBR1MCS-3-*welA*. The plasmid pET32a^(+)^ and *E. coli* BL21(DE3) were used to express the possible diguanylate phosphodiesterase encoded by Gene 1094. Both plasmids pJQ200SK and pBBR1MCS-3 were obtained from Wuhan Miaoling Bioscience and Technology Co., Ltd. Enzymes used in molecular cloning, including restriction enzymes, LA Taq DNA polymerase, and the DNA Ligation Kit were obtained from TaKaRa Biotechnology (Dalian, China). KOD FX DNA Polymerase was purchased from TOYOBO (shanghai) Biotech Co., Ltd. Primers were synthesized by Shanghai Sangon Biotechnology (Shanghai, China). All antibiotics used were obtained from Sigma Chemical Co. (St. Louis, MO, United States). Other reagents were obtained from Sinopharm Chemical Reagent Co., Ltd. (Shanghai, China) and Shanghai Macklin Biochemical Co., Ltd.

### Bioinformatic Analysis of WelA

Based on the amino acid similarity to GelA in gellan biosynthesis pathway (Harding et al., 2004), the gene *welA* (Gene 3707) was annotated on the whole genome of *Sphingomonas* sp. WG (GenBank no. LNOS00000000) (Li et al., 2016a) and its deduced protein WelA was obtained and analyzed by multiple bioinformatical tools. Firstly, the physical and chemical properties, hydrophilicity of WelA were predicted using ProtParam^1^ and ProtScale^2^ on the ExPASy Server (Wilkins et al., 1999). Furthermore, the related amino acid sequences were retrieved in the GenBank database using BlastP^3^ (Altschul et al., 1990) program, and the phylogenetic tree was constructed using ClustalX version 2.0 (Larkin et al., 2007) and the MEGA7 program (Kumar et al., 2016). The secondary structure prediction and domain architecture analysis were performed by SOPMA^4^ (Geourjon and Deleage, 1995) and SMART^5^ (Letunic et al., 2015), respectively.

### Construction of the Mutants △*welA* and the Complemented Strain

*welA* gene was knocked out from the chromosome of *Sphingomonas* sp. WG by double homologous recombination methods. Firstly, the upstream and downstream fragments (approximately 1,000 bp) of *welA* were amplified by PCR with primers welA5flFor/welA5flRev and welA3flFor/welA3flRev containing the tails with identity to the ends of each other (underlined) (Supplementary Table 1), respectively. Secondly, the obtained fragments were gel purified and fused into one DNA fragment with the help of their tails by fusing PCR reaction using “nested” primers welAdelFor/welAdelRev containing *Sac*I and *Xba*I recognition sites (underlined). Thirdly, the resulting fusion product was inserted into the *Sac*I-*Xba*I digested pJQ200SK to obtain the recombinant plasmid pJQ200SK-△*welA*. Finally, the pJQ200SK-△*welA* was transferred into *Sphingomonas* sp. WG from *E. coli* DH5α by triparental conjugal mating with the help of *E. coli* pRK2013. The single-crossover recombinants were screened by streptomycin and gentamicin. The deletion strains that undergo double-crossover recombination were screened on LB plates supplemented with 5% sucrose since single-crossover recombinants bearing the *sacB* gene are lethal in the presence of sucrose. The single-crossover recombinants were identified by PCR using two pairs of primers welA5flFor/welAinRev and welAinFor/welA3flRev, and the deletion strain △*welA* was confirmed by PCR using welAinFor/welAinRev as primers.

A *welA* fragment was inserted into the plasmid pBBR1MCS-3 to complement the *welA* gene. The ORF of *welA* was obtained by PCR using the *Sphingomonas* sp. WG genomic DNA as the template and welAexFor/welAexRev (The underlined parts were *Xho*I and *Xba*I recognition sties) as primers. Subsequentially, the gel-purified PCR product was digested by *Xho*I and *Xba*I and ligated into the linearized pBBR1MCS-3. The recombinant plasmid pBBR1MCS-3-*welA* was confirmed by colony PCR and DNA sequencing. Finally, the complemented strain of △*welA* with pBBR1MCS-3-*welA* was obtained by triparental conjugal mating and screened on LB supplemented with streptomycin and tetracycline. The complemented strain designated as △*welA*/pBBR1MCS-3-*welA* was further identified by colony PCR using the specific primers welAinFor/welAinRev for partial *welA* gene. The wild type containing the empty vector pBBR1MCS-3 named as WT/pBBR1MCS-3 was constructed through triparental conjugal mating using the method as described in our previous work (Li et al., 2021).

### Measurement of Cell Growth, Broth Viscosity, Exopolysaccharide Production of Different *Sphingomonas* sp. WG Strains

To elucidate the possible role of WelA in the EPS production, four different strains, wild-type of *Sphingomonas* sp. WG (WT), △*welA,△welA*/pBBR1MCS-3-*welA* and WT/pBBR1MCS-3 were cultivated in 50 mL fermentation medium in 250 mL shake flasks at 32.5°C, 200 rpm for different time. The fermentation parameters such as the biomass, the WL gum production, and the fermentation broth viscosity were measured as previously described (Li et al., 2018). The specific growth rate μ and time for the bacterial population to double (tD) were calculated according to the growth curve. The EPS production was determined with the phenol-sulfuric acid colorimetric method described previously using glucose as the standard (Zhou et al., 2017). The viscosity of fermentation broth was measured on a Brookfield Viscometer DV-III equipped with an ultra-low adaptor (Brookfield Engineering Laboratories) using the rotor spindle LV3 at 5 rpm at 25°C.

### Motility Assay of Different Strains in Semisolid Agar

The WT, △*welA*, and △*welA*/pBBR1MCS-3-*welA* strains were cultured in the LB medium at 30°C until OD_600_ was 0.6. The motility was tested on SIM media or fermentation medium supplemented with 0.3% (for swimming motility) and 0.5% (for swarming motility) of agar where 5 μL of bacterial suspension was dropped in each plate center and inoculated for 72 h (for swimming motility) and for 168 h (for swarming motility) at 28°C. The radius of circles formed by bacterial migration was measured to express the motility.

### RNA Extraction, cDNA Library Construction, Illumina Deep Sequencing, and Data Analysis

Cells of WT and △*welA* at the exponential phase (about 16 h of fermentation) were collected and harvested by centrifugation, respectively. Total RNA was extracted using RNAiso Plus (TaKaRa Biotechnology Company, China), and RNA integrity was assessed by an Agilent 2100 Bioanalyzer system (Agilent Technologies, CA, United States). RNA with a RIN value higher than 8.0 was chosen to produce a transcriptome library. According to the manufacturer’s instructions, cDNA libraries were generated using a NEBNext Ultra Directional RNA library prep kit for Illumina (NEB, United States). The cDNA library was sequenced on an Illumina sequencing platform (Illumina HiSeq™ 4000 platform) by the Shanghai Personal Biotechnology Co., Ltd. Library quality was assessed on an Agilent Bioanalyzer 2100 system.

Raw data (raw reads) in fastq format were filtered by removing low-quality reads, reads containing adapters, and ploy-N to obtain high-quality clean data used in all downstream analyses. The high-quality clean reads were mapped to the reference genome of *Sphingomonas* sp. WG (GenBank no. LNOS00000000) using Bowtie2-2.2.3 (Langmead and Salzberg, 2012). The level of gene expression was estimated by FPKM (Fragments Per Kilo bases per Million fragment). Differential expression analysis of the two groups (WT as the control and △*welA* as the experiment group) was performed using the DESeq (version 1.18.0) (Anders and Huber, 2010). Genes with a minimal twofold difference in expression (| log2 fold change (FC)| ≥ 1) and *p*-value < 0.05 were identified as differentially expressed genes (DEGs). Thus, the number of up-regulated and down-regulated genes was calculated based on the log_2_FC value.

DEGs with similar expression patterns were clustered by the Pheatmap R package using the Complete Linkage method. Furthermore, functional enrichment analysis was performed by Gene Ontology (GO) and KEGG enrichment analysis. For GO enrichment analysis, each DEG was mapped to GO terms in the Gene Ontology database^6^ by topGO (Alexa et al., 2006). The DEGs were also mapped to the KEGG orthology terms in the KEGG pathway database^7^ using the KOBAS software (Bu et al., 2021).

### Experimental Validation of Gene Expression by Quantitative Real-Time PCR (qRT-PCR)

The expressional levels of 10 typical DEGs identified by RNA-Seq were examined using qRT-PCR. Total RNA of WT and △*welA* cultured for 16 h were reverse-transcribed by a PrimeScript^®^ RT Reagent Kit with gDNA Eraser (Perfect Real Time) (TaKaRa, Dalian, China) according to the manufacturer’s instructions. qRT-PCR was performed using the primers listed in Supplementary Table 2 with a TB Green^®^Premix Ex Taq™ II (Tli RNaseH Plus) kit (TaKaRa, Dalian, China) on ABI 7500 instrument (Applied Biosystems). The qRT-PCR reaction system and conditions were the same as described in our previous work (Li et al., 2018). The relative expression of each gene was calculated according to the 2^–ΔΔ^ ^Ct^ formula, and the 16S rRNA gene served as an internal standard to standardize results. The expression of each gene was repeatedly detected at least three times. The data are expressed as arithmetic means ± the standard deviation.

### Quantitation of Intracellular c-di-GMP by HPLC-MS/MS

Firstly, WT, △*welA, and* △*welA/*pBBR1MCS-*welA* were cultured in fermentation media for about 12 h and harvested by centrifugation, respectively. Secondly, nucleotide extraction was carried out as previously reported (Newell et al., 2011) and was resuspended in 500 μL water under vigorous vortexing. Finally, the concentration of c-di-GMP was analyzed by reversed phase-coupled HPLC-MS/MS on a Waters Xevo TQ-S Triple Quad Mass Spectrometer using the method reported by Spangler et al. (2010).

Heterologous expression of possible diguanylate phosphodiesterase encoded by Gene 1094 in *E. coli* BL21 (DE3) and its activity assay.

Firstly, the recombinant expression plasmid was constructed. the ORF of Gene 1094 was amplified by PCR using the *Sphingomonas* sp. WG genomic DNA as the template and gene1094exFor/gene1094exRev containing *Bam*HI and *Hin*dIII recognition sties as primers (Supplementary Table 1). Sequentially, PCR product was gel-purified, digested by *Bam*HI and *Hin*dIII and ligated into the linearized pET32a^(+)^. The recombinant plasmid pET32a-1094 was confirmed by PCR using gene1094exFor/gene1094exRev as primers and DNA sequencing. Secondly, the recombinant plasmid was transformed into the *E. coli* BL21 (DE3) host to obtain the expression strain. Thirdly, the protein expression was induced by 0.8 mM isopropyl-D-thiogalactopyranoside (IPTG) at 28°C for 16 h in accordance with the pET system manual (Novagen, Germany). The cells untreated with IPTG was used as the control group. Then the crude enzyme samples were obtained by ultrasonic disruption of the cells, centrifugation and ultrafiltration. The samples were analyzed by sodium dodecyl sulfate–polyacrylamide gel electrophoresis (SDS-PAGE) to confirm the expression of the target protein.

Finally, the hydrolysis reaction of c-di-GMP was carried out in the mixture as follows: 50 mM Tris-HCl buffer (pH 9.35) containing 50 mM NaCl, 5 mM MgCl_2_ and 0.5 mM EDTA, 100 μM c-di-GMP as the substrate and the crude enzyme. The reaction was performed at 37°C for 2 h and was terminated by heating the samples at 100°C for 3 min. The precipitated proteins were removed by centrifugation at 16,000 × *g* and the obtained supernatant was filtered using a 0.22 μm membrane and analyzed by an HPLC instrument (Agilent 1260 Infinity II) equipped with a ZORBAX SB-C18 column (15 × 4.6 mm) and detected at 254 nm. The mobile phase A was 100 mM KH_2_PO_4_ containing 4 mM tetrabutylammonium hydrogen sulfate, and mobile phase B contained 75% phase A and 25% methanol. The phase B content in mobile phase was gradient from 0 to 30% from 2.5 to 5 min; 30 to 60% from 5 to 10 min; 60 to 100% from 10 to 14 min and at 100% for 7 min, then from 100 to 50% from 21 to 22 min and finally to 0 at 23 min. c-di-GMP was used as the standard. The enzyme activity (1 U) was defined as the amounts of enzyme required to hydrolyze 1 μM c-di-GMP per minute in 1 ml reaction mixture.

## Results

### Bioinformatical Analysis of WelA

One putative hybrid sensor histidine kinase (HK)/response regulator (RR) encoding gene *welA* was annotated in the genome of *Sphingomonas* sp. WG (Li et al., 2016a). The length of the open reading frame of *welA* was 2,385 bp, and therefore, the deduced protein WelA contained 794 amino acids. According to the predicted results of ProtParam, the calculated molecular mass and pI of WelA were 84.5 kDa and 5.14, respectively. The secondary structure prediction results by SOMPA showed the percentages of α-helix, extended strand, β turn, and random coil predicted were about 42.57, 18.14, 6.30, and 33.00. Structural domain analysis by SMART showed that this protein contains two PAS domains (residue 76–145 and residue 293–358), a HisKA domain (residue 418–484), a HATPase_c domain (residue 525–653), and a REC domain (residue 676–787), suggesting that this protein had both HK and RR domains and might be a hybrid sensor HK/RR. Besides, it also had two transmembrane regions (residue 15–34 and 41–63), indicating that it might be associated with the cell membrane.

**FIGURE 1:**
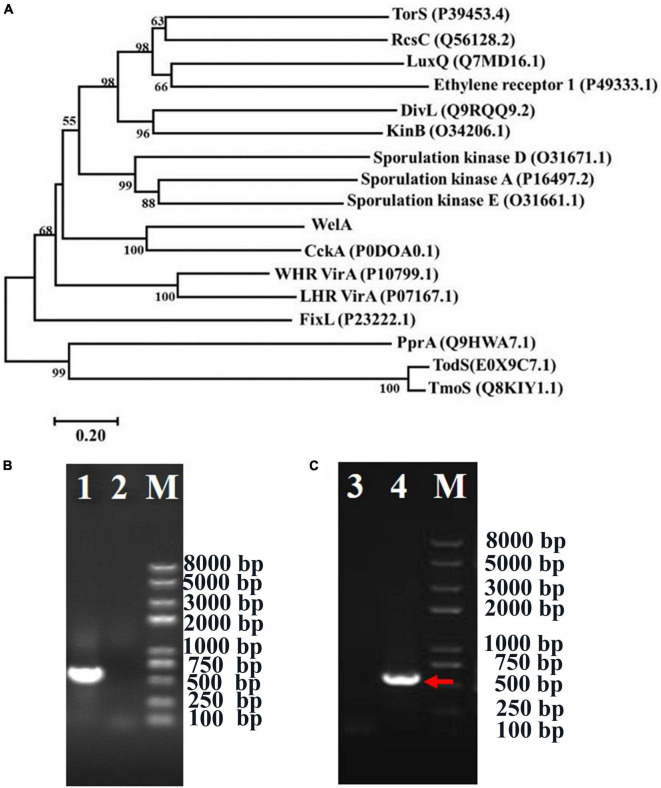
The phylogenetic analysis of WelA and verification of *welA* knock-out and complemented strain. **(A)** The phylogenetic tree of WelA based on the amino acid sequences. Sequence alignment was conducted by ClustalX2 software, and the tree was generated by MEGA 7 software using the neighbor-joining method. Bootstrap values, expressed as the percentages of 1,000 replications, are given at the branching points when they are higher than 50. Scale bar, 0.20 amino acid substitutions/site. **(B)** Identification of Δ*welA* mutant by PCR using primers welainfor/welainrev for partial *welA* encoding sequence. The PCR templates of different lanes were as follows: Lane 1: WT strain, Lane 2: Δ*welA* mutant. **(C)** Identification of the complemented strain by PCR using primers welAinFor and welAinRev for partial *welA* coding sequence. The PCR templates of different lanes were as follows: Lane 3: Δ*welA* mutant as negative control, Lane 4: complemented strain, M: D8000 DNA marker with the bands at 8,000, 5,000, 3,000, 2,000, 1,000, 750, 500, 250, and 100 bp. The right band was indicated by the arrow.

Subsequently, proteins homologous to WelA were retrieved from the Swissprot database by the BLASTp program. A phylogenetic tree was constructed by ClustalX multiple sequence alignment program version 2.0 (Larkin et al., 2007) and MEGA 7 program (Kumar et al., 2016; Figure 1A). WelA from *Sphingomonas* sp. WG showed 50.12% identity with the hybrid sensor HK CckA (Genbank no. P0DOA0.1) (Supplementary Figure 1) which initiated the conserved CckA–ChpT–CtrA–CpdR regulatory system controlling cell division, replication, and virulence of *Brucella abortus* (Willett et al., 2015). It also showed similarity to other sensor kinases such as DivL and FixL in varying degrees.

### The Effect of WelA on WL Gum Production

To investigate if WelA is involved in the regulation of WL gum biosynthesis, *welA* was deleted from the genome of *Sphingomonas* sp. WG by double homologous recombination using the method reported in our previous work (Li et al., 2021). The mutant strain Δ*welA* was verified by PCR with primers welAinFor and welAinRev (Figure 1B), and the specific product about 530 bp was lost. Besides, the *welA* coding sequence was introduced into Δ*welA* to obtain the complemented strain Δ*welA*/pBBR1MCS-3-*welA*, which was also elucidated by amplifying the partial *welA* coding sequence using welAinFor and welAinRev as primers (Figure 1C).

**FIGURE 2:**
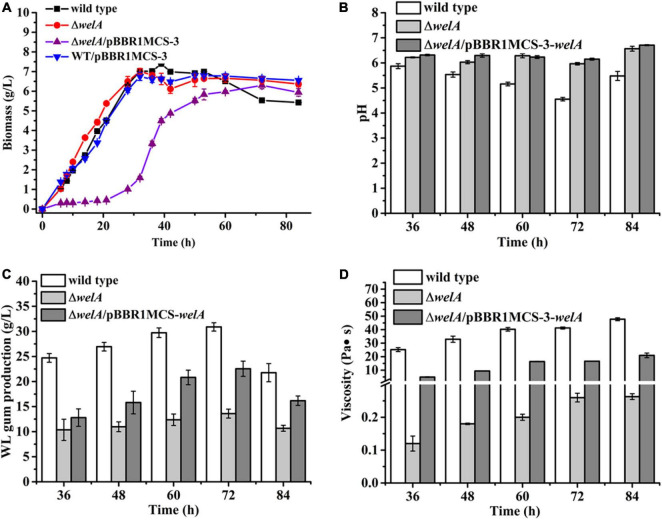
The fermentation results of WT, Δ*welA* mutant, and complemented strain △*welA*/pBBR1MCS-3-*welA*. **(A)** Comparison of biomass. **(B)** Comparison of pH. **(C)** Comparison of broth viscosity. **(D)** Comparison of WL gum production.

Subsequently, the fermentation of the WT, Δ*welA*, and Δ*welA*/pBBR1MCS-3-*welA* was performed (Figure 2). As the fermentation proceeded, the WT strain and the Δ*welA* strain entered the log phase or exponential phase very quickly (within 6 h), and their biomass increased significantly. Compared with WT strain, the deletion of *welA* did not reduce the cell growth rate, in contrast, it led to the enhanced growth rate. The μ_max_ for WT strain and Δ*welA* in the log phase was about 0.17 and 0.25 h^–1^, respectively. Δ*welA*/pBBR1MCS-3-*welA* showed a different growth curve in which it had a long lag phase for about 21 h and then entered the log phase with a μ about 0.12 h^–1^. The tD time for WT, Δ*welA*, and Δ*welA*/pBBR1MCS-3-*welA* was 4.26, 3.23, and 5.85 h, respectively. The slow specific growth rate of the complementary strain might be related to the replication burden of the plasmid pBBR1MCS-3-*welA* or the dose-dependent effect of the *welA* overexpression. Therefore, the growth curve of the other strain WT/pBBR1MCS-3 was determined. The empty vector had a very slight impact on the growth of the bacteria. Therefore, the slow growth rate of the complemented strain might be caused by a dose-dependent effect of the *welA* overexpression. Due to the influence of the metabolites such as organic acids, the pH of the fermentation broth changed. The pH value of WT was decreased and was the lowest among the three strains. The pH of the Δ*welA*/pBBR1MCS-3-*welA* was the highest, which might be related to its lowest biomass. Furthermore, the viscosity of the fermentation broth was detected as the accumulation of the EPS will enhance the viscosity. The viscosity of Δ*welA* fermentation broth was only 0.6% of WT, while the broth viscosity of the complemented strain could restore to 40% and 44% when cultured for 72 and 84 h, respectively. Similar to viscosity, *welA* regulated the WL gum biosynthesis, and the deletion of *welA* resulted in a remarkable decrease in WL gum production. The WL gum yield of Δ*welA* was only 44% and 49% of WT when cultured for 72 and 84 h. The gene complementation could restore the WL gum production to 73% and 74% of WT at 72 and 84 h. All these phenomena suggested that WelA plays important regulatory role in the WL gum biosynthesis process.

### Transcriptome Analysis of WT Strain and ΔwelA Mutant Strain

**FIGURE 3:**
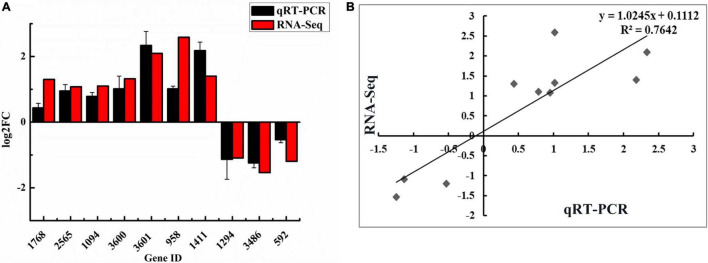
Validation of RNA-Seq results using qRT-PCR. **(A)** Comparison of the expression levels of ten DEGs detected by RNA-Seq and qRT-PCR method. **(B)** The correlation between RNA-Seq and qRT-PCR of the expression levels of ten DEGs.

Transcriptome analysis of WT (the control group) and Δ*welA* (the experiment group) was conducted to understand the genes differentially regulated by WelA. An average of 32,410,509 and 36,273,418 raw sequencing reads in the control group and experimental group was generated by Illumina sequencing, respectively. After the trimming process, about 93% and 90% of the libraries remained clean reads, respectively (Supplementary Table 3). The correlation of gene expression level between samples was checked to test the sequencing reliability of the experiment and the rationality of sample selection, and the results were shown in Supplementary Figure 2. The square of Pearson correlation coefficient (*R*^2^) between biological repeats was higher than 0.92 and suggested that the difference between individuals was very small and the sequence results were credible. Moreover, the six samples could be clustered into two groups, the WT and Δ*welA* group, which indicated that the deletion of *welA* resulted in a significant change of gene expression (Supplementary Figure 2). Therefore, differential expression analysis was performed. A total of 386 DEGs with 187 up-regulated genes and 199 down-regulated genes were detected (Supplementary Figure 3). To validate the accuracy of RNA-seq results, the expression of 10 typical DEGs that involved in signaling transduction pathway, sugar metabolism and WL gum biosynthesis, and c-di-GMP biosynthesis or hydrolysis was further confirmed by qRT-PCR. The results were shown in Figure 3. According to correlation analysis, the expression results of qRT-PCR were strongly positively consistent with those from RNA-Seq (*R*^2^ = 0.7642), which suggested that the data from the transcriptome were reliable.

### Gene Ontology (GO) and Kyoto Encyclopedia of Genes and Genomes (KEGG) Enrichment Analysis

**FIGURE 4:**
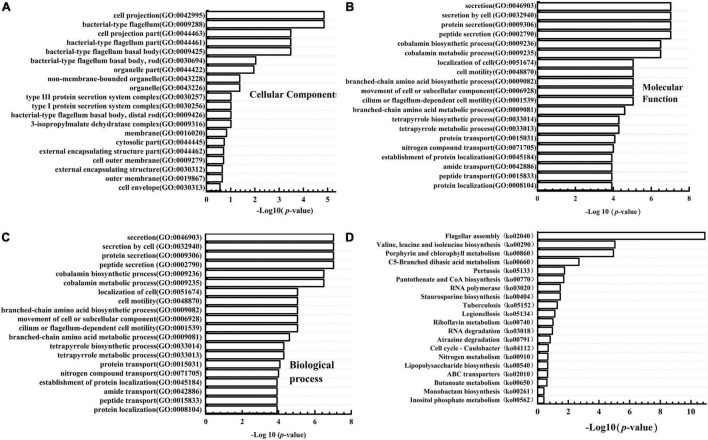
Gene ontology (GO) classification and Kyoto Encyclopedia of Genes and Genomes (KEGG) pathway enrichment analysis of the differentially expressed genes in Δ*welA*. **(A)** The DEGs categorized based on Cell component; **(B)** the DEGs categorized based on Molecular function; **(C)** the DEGs categorized based on biological process. **(D)** KEGG pathway enrichment analysis of DEGs.

GO enrichment analysis was performed to gain the information of DEGs functional categories. The results (Figure 4) showed that the “bacterial-type flagellum (GO:0009288)” and “cell projection (GO:0042995)” were the top GO terms in the “cellular component” group; The “methyltransferase activity (GO:0008168)” and “transferase activity, transferring one-carbon groups (GO:0016741)” were significantly enriched in “molecular function” group. The “secretion (GO:0046903),” “secretion by cell (GO:0032940),” “protein secretion (GO:0009306),” and “peptide secretion (GO:0002790)” were significantly more clustered in the “biological process category.” Besides, the DEGs were also mapped to different KEGG categories, including cellular processes, environmental information processing, genetic information processing, human diseases, and metabolism. The top 20 enriched pathways were also shown in Figure 4. The most DEGs were enriched in “Flagellar assembly (ko02040),” “Porphyrin and chlorophyll metabolism (ko00860),” “Valine, leucine, and isoleucine biosynthesis (ko 00290),” “C5-Branched dibasic acid metabolism (ko00660).” Since we focused on the role of WelA on the biosynthesis of WL gum, the regulation pathway of WelA was mainly analyzed in the following step.

### DEGs in Two-Component Signal Transduction Systems

WelA was mapped to the two-component signal transduction systems (ko02020) due to its identity to CckA, and its deletion resulted in the up-regulated expression of *chpT* (Gene 3285) and *ctrA* (Gene 1411) (Table 1). It has been reported that CtrA is an active transcriptional regulator controlling genes involved in cell cycle progression and flagellar motility in *Caulobacter crescentus* (Laub et al., 2000). Besides CtrA, CpdR (Gene 1015) is another RR that can also be phosphorylated by ChpT. Normally, CpdR inhibits CtrA, and this inhibition effect could be relieved by phosphorylation. However, the expression level of CpdR was not significantly changed after *welA* deletion. In general, genes controlled by CtrA are enriched in certain functional categories, i.e., cell motility, signal transduction, and cell wall/membrane envelope biogenesis (Brilli et al., 2010). In *C. crescentus*, the architecture of the CtrA promoters has been analyzed, and two classes of CtrA binding motifs: a full site represented by the sequence TTAA (N_7_) TTAA, and a half site (TTAA) were revealed (Zhou et al., 2015). It has been reported that the expression of the fla2 genes involved in the biosynthesis of polar flagella of *Rhodobacter sphaeroides* is dependent on the two-component system, including CckA-ChpT-CtrA (Vega-Baray et al., 2015). Based on the motif sequence scanning, CtrA might regulate the expression of many genes. The expression of two special genes involved in c-di-GMP hydrolysis and flagellar assembly was up-regulated by CtrA. The first gene was Gene1094 encoding the diguanylate phosphodiesterase that might be related to hydrolysis of c-di-GMP. The heterologous expression of Gene 1094 in *E. coli* BL21 (DE3) showed that the crude enzyme had weak activity to catalyze the hydrolysis of c-di-GMP (Supplementary Figure 4). Its highly expression level indicated that c-di-GMP level might decreased and was involved in the regulation of WL gum biosynthesis. Therefore, the intracellular c-di-GMP was extracted and determined. As expected, the intracellular c-di-GMP concentration was significantly decreased in Δ*welA* (Supplementary Figure 5). Generally, reduced c-di-GMP level might increase flagellar motility and decrease the biofilm formation (Floyd et al., 2020). The second one is FlgN (Gene 283), which is the flagellar synthesis and assembly chaperone, and therefore, the motility might be changed.

**TABLE 1:** The expression level of genes in two-component system and in WL gum biosynthesis.

Gene ID	Gene product description	Log_2_FC	*p*-value
**DEGs in two-component system**			
Gene3285	Histidine phosphotransferase ChpT	1.12	0.0036
Gene1411	Cell cycle transcriptional regulator CtrA	1.40	1.86E-09
Gene1015	Two-component system cell cycle RR CpdR	0.67	0.0087
Gene1094	Diguanylate phosphodiesterase	1.10	0.026
Gene283	Flagellar protein FlgN	1.09	0.0053
Gene1533	C4-dicarboxylate transport protein DctA	1.88	2.55E-05
Gene 3280	Chemotaxis protein CheR	-1.01	0.0023
Gene 371	Cytochrome C	-1.44	0.025
**Genes in WL gum Biosynthesis**			
Gene ID	Gene product	Log_2_FC	*p*-value
Gene3585	WelG	0.21	0.31
Gene3586	WelS	0.17	0.50
Gene3587	WelR	0.26	0.23
Gene3588	WelQ	0.34	0.16
Gene3589	WelI	0.08	0.74
Gene3590	Welk	-0.11	0.58
Gene3591	WelL	-0.41	0.075
Gene3592	WelJ	0.23	0.33
Gene3593	Hypothetical protein	0.48	0.17
Gene3594	WelF	0.33	0.17
Gene3595	WelD	0.56	0.021
Gene3596	WelC	0.47	0.036
Gene3597	WelE	0.45	0.063
Gene3598	WelM	0.26	0.25
Gene3599	WelN	-0.64	0.0084
Gene3600	AtrD	1.32	0.00033
Gene3601	AtrB	2.09	1.1E-05
Gene3602	Hypothetical protein	-0.25	0.30
Gene3603	WelB	0.67	0.0031
Gene3604	RmlA	0.12	0.64
Gene3605	RmlC	0.38	0.24
Gene3606	RmlB	-0.08	0.87
Gene3607	RmlD	-0.74	0.0016
Gene 896	PGM	0.65	0.094
Gene3404	UGP	0.53	0.026
Gene3147	UGD	0.63	0.031
Gene592	PMM	-1.19	5.6E-06

Besides the CckA-ChpT-CtrA pathway, the other three genes were mapped to the two-component signal transduction systems (ko02020), including the up-regulated DctP (Gene1533) in the C4-Dicarboxyate transport, the down-regulated CheR (Gene 3280) in flageller assembly, and Cyt C (Gene 371) in electron transfer system in Oxidative phosphorylation process.

### Genes Involved in WL Gum Biosynthesis

Many genes were involved in the biosynthesis of WL gum. However, most genes related to the biosynthesis of nucleotide-sugar precursors such as *pgm*, *ugp*, *ugd*, and *rlmA* to *rmlD*, the tetrasaccharide repeating unit assembly such as *welB*, *welK*, *welL*, and *welQ*, polymerization of the assembled repeating units such as *welS*, *welG*, *welE*, *welC*, etc., were not identified as DEGs (Table 1). The expressional level of gene encoding PMM (Gene 592) catalyzing the synthesis of α-D-mannose 1-phosphate, the precursor of GDP-mannose, was down-regulated (the log2FC was -1.19). Besides, two genes (Gene 3600 and 3601) encoding AtrD and AtrB in the *wel* cluster were identified as up-regulated DEGs.

### Regulation of Flagella Synthesis and Motility of WelA

**FIGURE 5:**
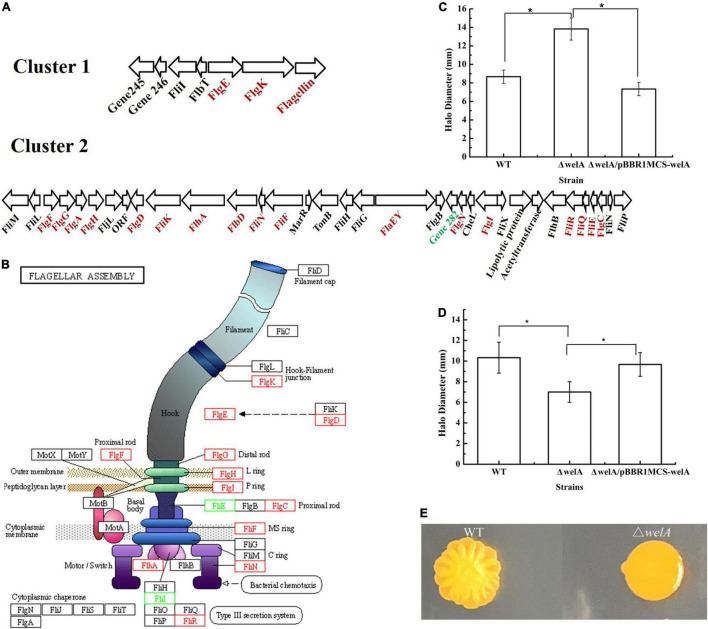
The expressional levels of genes involved in flagellar assembly and effects on motility. **(A)** Cluster I and II involved in flagellar assembly. Highly expressed genes are red and lowly expressed genes are green. **(B)** The Schematic diagram of the differentially expressed genes involved in flagellar assembly (KEGG pathway ko02040). Highly expressed genes are red and lowly expressed genes are green. **(C)** Swimming motility on SIM medium. **(D)** Swimming motility on solid fermentation medium. **(E)** Swarming motility. * means *p* < 0.05.

According to the GO enrichment results (Figure 4), the DEGs under the category of biological process related to processes of flagellum-dependent cell motility (GO:0001539) and under the categories of the cellular component involved in bacterial-type flagellum (GO:0009288) were highly expressed. KEGG pathway analysis revealed that 14 DEGs were involved in flagellar biosynthesis, including 12 highly and 2 lowly expressed genes. Taken together, WelA might play a regulatory role in flagellar biosynthesis of *Sphingomonas* sp. WG. Therefore, the organization of the genes for flagellar biosynthesis was analyzed. Most of the genes were clustered together in the smaller cluster I and the larger cluster II within the chromosome, as shown in Figure 5 and Supplementary Table 4. Most of the highly expressed genes in Δ*welA* encode essential components for flagellar biosynthesis, including flagellin, flagellar basal body protein, flagellar hook-associated protein, and export apparatus for flagellar components. Furthermore, three genes that encode transcriptional regulatory proteins FlbD, FlaEY, and FlaF were also highly expressed. However, some genes in the chemosensory system that also influence motility, such as CheR, McpA, and twitching motility protein PilT, were down-regulated.

Therefore, it is inferred that the flagellar formation and the motility of the mutant strain might change. Therefore, the swimming and swarming motilities of WT and *welA* mutant strain were measured. Δ*welA* showed higher swimming motility than WT on SIM medium (Figure 5C); however, when cultured on solid fermentation medium, its swimming motility was much lower (Figure 5D). Its swarming motility independent of the presence of a flagellum was lost on both media (Figure 5E).

## Discussion

In prokaryotes, two-component signal transduction systems are the predominant means to respond to a wide range of external stimuli such as antibiotics and quorum-sensing signals. Normally, each two-component signal transduction system consists of a sensor HK protein that can be autophosphorylated and a RR protein with the N-terminal receiver domain (REC) receiving the phosphoryl group required for activation of the regulatory domain. Through the signal transduction, many physiological changes will be triggered by changing gene expression programs, altering swimming behavior etc. (Skerker et al., 2005; Mattos-Graner and Duncan, 2017). As a hybrid sensor HK/RR, besides HisKA and REC domain, WelA also contained two common conserved signal input PAS domains suggesting that the protein has a “sensor” capacity (Christensen and Serbus, 2015). Therefore, WelA might play important roles in WL gum biosynthesis and other cellular physiology. As expected, WelA affected the WL gum production and fermentation broth viscosity through gene deletion manipulation. Through KEGG enrichment analysis, WelA was mapped to CckA in the branched CckA-ChpT-CtrA phosphorelay pathway. The deletion of *welA* resulted in the hyperactive expression of ChpT and CtrA. Similarly, the deletion of *cckA* in *Rhodobacter capsulatus* resulted in increased expression of ChpT and CtrA (Koppenhofer and Lang, 2020). Besides CckA, ChpT might also receive signals from other kinase and transduce them to CtrA (Koppenhofer and Lang, 2020). The activity of CtrA was also higher in *Sphingomonas melonis* Δ*cckA* than that of WT strain, which might be due to the absence of CckA phosphatase activity (Francez-Charlot et al., 2015). CtrA is a master regulator of motility and regulates EPS synthesis and cyclic-di-GMP signaling in *S. melonis* (Francez-Charlot et al., 2015). Thus, the more expressed ChpT and CtrA might influence many cellular metabolisms. One carbon metabolism was critical for DNA methylation and DNA synthesis (Lee et al., 2009) because it plays an important role in the formation of many methyl donors such as S-adenosylmethionine and donors that could be recognized by DNA, RNA, histone, and protein methyltransferases (Lyon et al., 2020). DNA methylation regulated many cellular processes such as protein–protein interactions, DNA replication initiation and gene expression in bacteria (Sánchez-Romero and Casadesús, 2020). Therefore, the enriched genes in “transferase activity, transferring one-carbon groups (GO:0016741)” might involve in the gene expression regulation in *welA* mutant strain.

Firstly, the higher expressional level of genes involved in the flagellar assembly in *welA* mutant strain was detected. Many genes are required for the bacterial flagellum assembly, such as structural subunits, regulatory proteins, motor proteins, export apparatus for flagellar components, and the chemosensory machinery (Aldridge and Hughes, 2002). In many bacterial species, the expression of the flagellar genes was regulated in a hierarchical pattern in which genes might be classified into III classes and IV classes in some cases. For example, the transcriptional activator FlhD/FlhC, the sigma factor FliA (δ^28^), and its specific anti-sigma factor FlgM regulated the hierarchical expression of flagellar genes in *E. coli* and *Salmonella enterica* (Rivera-Osorio et al., 2018). The FlhD and FlhC activated the genes for the hook and basal body assembly and FliA with the help of the sigma factor (δ^70^). When the basal body and the hook are assembled, the exportation of FlgM is occurred, which resulted in the association of FliA with the core RNA polymerase to recognize the promoters of genes to facilitate filament formation and chemotaxis (Maruyama et al., 2015). In several species, the expression of the early flagellar genes is induced by sigma factor RpoN and an activator protein, while the late genes are regulated by FliA and FlgM (Smith and Hoover, 2009; Tsang and Hoover, 2014). In *C. crescentus* and *Sinorhizobium meliloti*, the expression of several genes involved in flagellar biosynthesis is directly activated by the transcriptional regulator CtrA (Pini et al., 2015; Rivera-Osorio et al., 2018). However, there is little information about flagellar biosynthesis and motility of Sphingomonads. In *Sphingomonas wittichii*, the genes involved in the biosynthesis and assembly of flagellar are in three chromosomal regions (Swit_0212-0213, Swit_1260-1293, and Swit_1458) and its biosynthesis might be regulated by a putative Fli-type RNA polymerase sigma-28 factor (Swit_1281). Furthermore, three genes encoding pili assembly proteins (Swit_0565, Swit_0615, and Swit_0616) were also characterized (Johnson et al., 2011). Two types (lateral and polar) of flagellar gene sets were found in *Sphingomonas* sp. strain A1, although it has a single polar flagellum, and the set II clusters contained the regulatory protein-encoding genes *flhC*, *flhD* and *fliA*. However, the regulation mechanism was unclear because no common motif for FlhD2C2 binding was found in its genome (Maruyama et al., 2015).

The organization of genes for flagellar biosynthesis in *Sphingomonas* sp. WG was similar to that of the sphingan*-*producing *Sphingomonas* sp. strain S2M10 (Czieborowski et al., 2020). However, it was different from that in *Sphingomonas melonis* (Francez-Charlot et al., 2015) and *Sphingomonas* A1 (Maruyama et al., 2015). The commonly regulatory protein-encoding gene such as *flhC*, *flhD and fliA* was not found in clusters I and II. It seemed that the hierarchical regulation pattern of flagellar biosynthesis in *Sphingomonas* sp. WG was similar to *C. crescentus* with the regulatory proteins CtrA, the δ^54^ transcriptional activator FlbD and its *trans*-acting regulator FliX, the repressor protein FlbT and post-transcriptional regulator FlaF, and the possible regulatory protein FlaEY. Genes encoding FlbD, FliX, FlbT, and FlaEY were in the cluster, and those encoding FlaF and CtrA were in other loci of the genome. The expressional level of *flbT and fliX* was not changed significantly. However, *ctrA, flbD, flaEY*, and *flaF* were up-regulated. CtrA might regulate the expression of *flgN*, which encodes the flagellar synthesis and assembly chaperones through the binding motif screening. Besides FlgN, three proteins FlhA, FliQ, and FliR were also up-regulated, which belong to the transmembrane export gate that acts as a proton/protein antiporter in the export machinery. The enhanced expression of FlgN, FlhA, FliQ, and FliR might be beneficial for the export machinery to transport the structural subunits of flagellar from the cytoplasm to the growing flagellar distal end (Minamino et al., 2021). FlbD and FliX tightly controlled the expression of the genes involved in the assembly of LP-ring, distal rod, and hook of the subpolar flagellum, and the extracellular polysaccharide production and biofilm formation in *Bradyrhizobium diazoefficiens* (Dardis et al., 2021). FlaE and FlaY have been proved to modulate the expression level of the flagellin genes and several genes encoding chemotaxis functions in *C. crescentus* (Minnich et al., 1988). In *Sphingomonas* sp. strain S2M10, the mutant of FlaQ, the homolog of FlaEY, was proved to enhance the expression of *fliC* and reduce the expression of *flgI* and a hypothetical protein (Czieborowski et al., 2020). Therefore, the more expressed master regulators might participate in the regulation of flagellar biogenesis; however, the flagellar regulatory cascade remains unclear.

Moreover, the changes of flagellar were related to the motility of the cells. For most bacteria, flagella are the major organelles responsible for swimming motility and type IV pili (TFP) are responsible for the twitching motility. The swimming motility might be related to the length of flagellum. When the FlaC was knocked out, short flagellum was formed and the swimming motility was decreased (Jung et al., 2021). In our work, we found that the swimming motility of the mutant stain was dependent on the medium, which might be related to the complicated regulation mechanism of flagellar formation. In the other respect, the swarming motility of *Pseudomonas aeruginosa* is dependent on flagella, TFP and presence of rhamnolipids (Overhage et al., 2007). Therefore, the down-regulated twitching motility protein PilT, an ATPase responsible for TFP, might lead to the decreased swarming motility.

The expressional levels of genes involved in sphingan WL gum production must also be given attention. Most genes in the biosynthesis pathway were not identified as DEGs. Similar results were observed in *Sphingomonas* sp. ATCC 31555. The expressional level of ten genes in welan gum biosynthesis was not changed between the inorganic nitrogen group, organic nitrogen group, and inorganic-organic combined nitrogen group, although their welan gum production was different (Xu et al., 2017). In Δ*welA*, only three related genes encoding PMM, AtrB, and AtrD were DEGs. AtrB and AtrD showed similarity to several ABC-type transporter proteins from diverse genera of bacteria. They were mapped as HlyB/CyaB and HlyD/CyaD in the type I secretion system. However, the function of AtrB and AtrD in WL gum biosynthesis was unknown. ABC transporters are important components of a large class of proteins involved in many biological processes, such as uptake of nutrients and export of proteins, drugs, and capsular polysaccharides in many Gram-negative bacteria (Willis and Whitfield, 2013). The over-expression of AtrB and AtrD resulted in significant enhancement of WL gum production of *Sphingomonas* sp. WG (Data unpublished). Therefore, the enhanced AtrB and AtrD might be related to the WL gum biosynthesis in an unknown way. Besides, the lower expression of phosphomannomutase encoding gene *pmm* might lead to less GDP-mannose precursor formation, and thereby less WL gum biosynthesis. Furthermore, the regulation of EPS was at the mRNA level and at the protein level. The over-expression of GelA (the homologous of WelA) enhanced Gellan gum (Lee et al., 2017) and the EPS produced by *Sphingobium chungbukense* DJ77, and resulted in the up-regulated EPS export protein and one unknown protein and the down-regulated putative glucose dehydrogenase and bifunctional acetyltransferase. As the critical components for biofilm formation or virulence, many extracellular polysaccharides are directly or indirectly activated by the second massager cyclic-di-GMP, which plays important role in the intercellular signal transduction (Schmid et al., 2018). The intracellular concentration of this c-di-GMP can be modulated by the synthesis and degradation catalyzed by diguanylate cyclases and specific phosphodiesterases under environmental and physiological cues through complex signal transduction systems. Generally, high concentrations of c-di-GMP promote biofilm formation, including extracellular polysaccharides and other matrix components biosynthesis. c-di-GMP binds to specific effector proteins such as Alg44 with PilZ domain, FimX with the EAL domain, PopA with RxxD motif, and Clp or the responsive riboswitch (GEMM motif) (Boyd and O’Toole, 2012). The expression of one possible phosphodiesterase regulated by CtrA was enhanced. Therefore, the intracellular c-di-GMP was decreased in Δ*welA*. The lower c-di-GMP might involve in the WL gum biosynthesis and lead to the lower WL gum production. Notably, the possible effectors of c-di-GMP were not clear in the strain *Sphingomonas* sp. WG although its effectors of the other kinds of polysaccharides were investigated before. Therefore, the signal transduction pathway of c-di-GMP will be investigated in next experiment.

Both EPSs and motility are significant contributing factors in biofilm formation. Sphingan-dependent biofilm formation of *Sphingomonas* sp. strain S2M10 was observed in the presence of specific carbon sources (Czieborowski et al., 2020). Biofilm formation is a stepwise process. For the early stages of biofilm formation, cells transited from motile to a non-motile state for a reversible surface attachment. Then it might form multicellular aggregates based on the swarming motility and the production of EPS such as sphingans to establish the stable association with the substratum. Swarming motility did affect not only the formation but also the architecture of the biofilm. For example, for *P. aeruginosa*, hyper warming motility led to a flat and uniform biofilm, whereas lower swarming motility resulted in microcolonies (Wang et al., 2014). Therefore, WelA might influence the formation of biofilm of *Sphingomonas* sp. WG due to the lost swarming motility and lower EPS production.

## Conclusion

**FIGURE 6:**
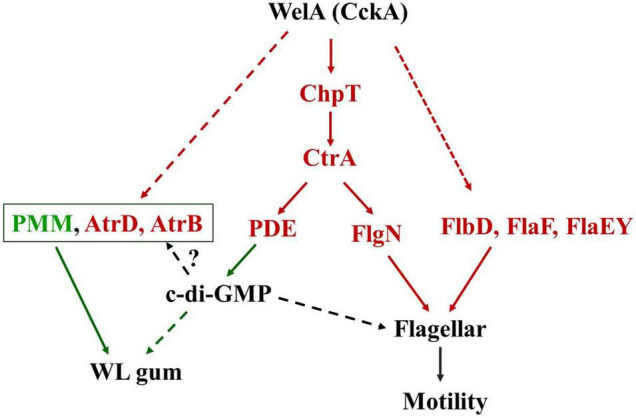
A tentative model for the WelA regulatory circuit to regulate the WL gum biosynthesis and flagellar motility. The solid lines indicate the potential phosphate flow from CckA to CtrA via ChpT or activation of regulatory proteins. The dashed lines indicate the unknown regulatory pathway. Proteins in red color were up-regulated whereas those in green color were down-regulated. The red arrows indicated that the interactions result in activation while the green one indicated the inhibiting interactions.

In conclusion, it was obvious that WelA contributes to WL gum biosynthesis and motility of *Sphingomonas* sp. WG through the branched CckA-ChpT-CtrA pathway and c-di-GMP signaling pathway. A model that features the possible role of WelA in *Sphingomonas* sp. WG was shown in Figure 6. Therefore, the functions of genes in the CckA-ChpT-CtrA pathway and c-di-GMP signaling pathway will be identified in our lab, which will provide more information on the regulation of WL gum biosynthesis and be beneficial for the construction of the WL gum highly producing strains by metabolic engineering.

## Data Availability Statement

The datasets presented in this study can be found in online repositories. The names of the repository/repositories and accession number(s) can be found in the article/Supplementary Material.

## Author Contributions

HL and MC: writing—original draft, writing—review and editing, conceptualization, data curation, and formal analysis. ZZ and BL: methodology and data curation. JL and HX: writing—review and editing, investigation, and data curation. SJ and ZG: investigation and data curation. JW and HZ: conceptualization, supervision, and project administration. All authors contributed to the article and approved the submitted version.

## Conflict of Interest

The authors declare that the research was conducted in the absence of any commercial or financial relationships that could be construed as a potential conflict of interest.

## Publisher’s Note

All claims expressed in this article are solely those of the authors and do not necessarily represent those of their affiliated organizations, or those of the publisher, the editors and the reviewers. Any product that may be evaluated in this article, or claim that may be made by its manufacturer, is not guaranteed or endorsed by the publisher.

## Footnotes

1
http://web.expasy.org/protparam/

2
http://web.expasy.org/protscale/

3
http://blast.ncbi.nlm.nih.gov/Blast.cgi

4
https://npsa-prabi.ibcp.fr/cgi-bin/npsa_automat.pl?page=/NPSA/npsa_sopma.html

5
http://smart.embl-heidelberg.de/

6
http://www.geneontology.org/

7
http://www.kegg.jp/kegg/kegg1.html

## References

[B1] Aldridge P. Hughes K. T. (2002). Regulation of flagellar assembly. *Curr. Opin. Microbiol.* 5 160–165. 10.1016/S1369-5274(02)00302-8 11934612

[B2] Alexa A. Rahnenführer J. Lengauer T. (2006). Improved scoring of functional groups from gene expression data by decorrelating GO graph structure. *Bioinformatics* 22 1600–1607. 10.1093/bioinformatics/btl140 16606683

[B3] Altschul S. F. Gish W. Miller W. Myers E. W. Lipman D. J. (1990). Basic local alignment search tool. *J. Mol. Biol.* 215 403–410. 10.1016/S0022-2836(05)80360-2 2231712

[B4] Anders S. Huber W. (2010). Differential expression analysis for sequence count data. *Genome Biol.* 11:R106. 10.1186/gb-2010-11-10-r106 20979621 PMC3218662

[B5] Boyd C. D. O’Toole G. A. (2012). Second messenger regulation of biofilm formation: breakthroughs in understanding c-di-GMP effector systems. *Annu. Rev. Cell Dev. Biol.* 28 439–462. 10.1146/annurev-cellbio-101011-155705 23057745 PMC4936406

[B6] Brilli M. Fondi M. Fani R. Mengoni A. Ferri L. Bazzicalupo M. (2010). The diversity and evolution of cell cycle regulation in alpha-*proteobacteria*: a comparative genomic analysis. *BMC Syst. Biol.* 4:52. 10.1186/1752-0509-4-52 20426835 PMC2877005

[B7] Bu D. Luo H. Huo P. Wang Z. Zhang S. He Z. (2021). KOBAS-i: intelligent prioritization and exploratory visualization of biological functions for gene enrichment analysis. *Nucleic Acids Res.* 49 W317–W325. 10.1093/nar/gkab447 34086934 PMC8265193

[B8] Christensen S. Serbus L. R. (2015). Comparative analysis of Wolbachia genomes reveals streamlining and divergence of minimalist two-component systems. *G3* 5 983–996. 10.1534/g3.115.017137 25809075 PMC4426382

[B9] Coleman R. J. Patel Y. N. Harding N. E. (2008). Identification and organization of genes for diutan polysaccharide synthesis from Sphingomonas sp. ATCC 53159. *J. Ind. Microbiol. Biotechnol.* 35 263–274. 10.1007/s10295-008-0303-3 18210176

[B10] Czieborowski M. Hubenthal A. Poehlein A. Vogt I. Philipp B. (2020). Genetic and physiological analysis of biofilm formation on different plastic surfaces by *Sphingomonas* sp. strain S2M10 reveals an essential function of sphingan biosynthesis. *Microbiology* 166 918–935. 10.1099/mic.0.000961 32762802

[B11] Dardis C. Quelas J. I. Mengucci F. Althabegoiti M. J. Lodeiro A. R. Mongiardini E. J. (2021). Dual control of flagellar synthesis and exopolysaccharide production by FlbD-FliX class II regulatory proteins in *Bradyrhizobium diazoefficiens*. *J. Bacteriol.* 203 e00403–e00420. 10.1128/JB.00403-20 33468586 PMC8088514

[B12] Fialho A. M. Moreira L. M. Granja A. T. Popescu A. O. Hoffmann K. Sa-Correia I. (2008). Occurrence, production, and applications of gellan: current state and perspectives. *Appl. Microbiol. Biotechnol.* 79 889–900. 10.1007/s00253-008-1496-0 18506441

[B13] Floyd K. A. Lee C. K. Xian W. Nametalla M. Valentine A. Crair B. (2020). c-di-GMP modulates type IV MSHA pilus retraction and surface attachment in *Vibrio cholerae*. *Nat. Commun.* 11:1549. 10.1038/s41467-020-15331-8 32214098 PMC7096442

[B14] Francez-Charlot A. Kaczmarczyk A. Vorholt J. A. (2015). The branched CcsA/CckA-ChpT-CtrA phosphorelay of *Sphingomonas melonis* controls motility and biofilm formation. *Mol. Microbiol.* 97 47–63. 10.1111/mmi.13011 25825287

[B15] Freitas F. Alves V. D. Reis M. A. (2011). Advances in bacterial exopolysaccharides: from production to biotechnological applications. *Trends Biotechnol.* 29 388–398. 10.1016/j.tibtech.2011.03.008 21561675

[B16] Geourjon C. Deleage G. (1995). SOPMA: significant improvements in protein secondary structure prediction by consensus prediction from multiple alignments. *Comput. Appl. Biosci.* 11 681–684. 10.1093/bioinformatics/11.6.681 8808585

[B17] Harding N. E. Patel Y. N. Coleman R. J. (2004). Organization of genes required for gellan polysaccharide biosynthesis in Sphingomonas elodea ATCC 31461. *J. Ind. Microbiol. Biotechnol.* 31 70–82. 10.1007/s10295-004-0118-9 14767675

[B18] Johnson D. R. Coronado E. Moreno-Forero S. K. Heipieper H. J. van der Meer J. R. (2011). Transcriptome and membrane fatty acid analyses reveal different strategies for responding to permeating and non-permeating solutes in the bacterium *Sphingomonas wittichii*. *BMC Microbiol.* 11:250. 10.1186/1471-2180-11-250 22082453 PMC3238334

[B19] Jung Y. C. Lee M. A. Kim H. S. Lee K. H. (2021). Role of DegQ in differential stability of flagellin subunits in *Vibrio vulnificus*. *Npj Biofilms Microbiomes* 7:32. 10.1038/s41522-021-00206-7 33833236 PMC8032703

[B20] Koppenhofer S. Lang A. S. (2020). Interactions among Redox Regulators and the CtrA Phosphorelay in *Dinoroseobacter shibae* and *Rhodobacter capsulatus*. *Microorganisms* 8:562. 10.3390/microorganisms8040562 32295208 PMC7232146

[B21] Kovach M. E. Elzer P. H. Hill D. S. Robertson G. T. Farris M. A. Roop R. M. II (1995). Four new derivatives of the broad-host-range cloning vector pBBR1MCS, carrying different antibiotic-resistance cassettes. *Gene* 166 175–176. 10.1016/0378-1119(95)00584-1 8529885

[B22] Kumar A. S. Mody K. Jha B. (2007). Bacterial exopolysaccharides–a perception. *J. Basic Microbiol.* 47 103–117. 10.1002/jobm.200610203 17440912

[B23] Kumar S. Stecher G. Tamura K. (2016). MEGA7: molecular evolutionary genetics analysis version 7.0 for bigger datasets. *Mol. Biol. Evol.* 33 1870–1874. 10.1093/molbev/msw054 27004904 PMC8210823

[B24] Langmead B. Salzberg S. L. (2012). Fast gapped-read alignment with Bowtie 2. *Nat. Methods* 9 357–359. 10.1038/nmeth.1923 22388286 PMC3322381

[B25] Larkin M. A. Blackshields G. Brown N. P. Chenna R. McGettigan P. A. McWilliam H. (2007). Clustal W and Clustal X version 2.0. *Bioinformatics* 23 2947–2948. 10.1093/bioinformatics/btm404 17846036

[B26] Laub M. T. McAdams H. H. Feldblyum T. Fraser C. M. Shapiro L. (2000). Global analysis of the genetic network controlling a bacterial cell cycle. *Science* 290 2144–2148. 10.1126/science.290.5499.2144 11118148

[B27] Lee S. Y. Ahn J. Y. Kim M. Sekhon S. S. Cho S. J. Kim Y. C. (2017). Phenotypic and proteomic analysis of positively regulated gellan biosynthesis pathway in *Sphingomonas elodea*. *Anim. Cells Syst.* 21 115–123. 10.1080/19768354.2017.1290678 30460059 PMC6138312

[B28] Lee Y. L. Xu X. R. Wallenstein S. Chen J. (2009). Gene expression profiles of the one-carbon metabolism pathway. *J. Genet. Genomics* 36 277–282. 10.1016/S1673-8527(08)60115-0 19447375 PMC2684624

[B29] Lelchat F. Cozien J. Le Costaouec T. Brandilly C. Schmitt S. Baudoux A. C. (2015). Exopolysaccharide biosynthesis and biodegradation by a marine hydrothermal *Alteromonas* sp. strain. *Appl. Microbiol. Biotechnol.* 99 2637–2647. 10.1007/s00253-014-6075-y 25319363

[B30] Letunic I. Doerks T. Bork P. (2015). SMART: recent updates, new developments and status in 2015. *Nucleic Acids Res.* 43 D257–D260. 10.1093/nar/gku949 25300481 PMC4384020

[B31] Li H. Jiao X. Sun Y. Sun S. Feng Z. Zhou W. (2016b). The preparation and characterization of a novel sphingan WL from marine Sphingomonas sp. WG. *Sci. Rep.* 6:37899. 10.1038/srep37899 27883073 PMC5121650

[B32] Li H. Feng Z. M. Sun Y. J. Zhou W. L. Jiao X. Zhu H. (2016a). Draft Genome sequence of *Sphingomonas* sp. WG, a Welan gum-producing strain. *Genome Announc.* 4:e01709-15. 10.1128/genomeA.01709-15 26868397 PMC4751321

[B33] Li H. Li J. Jiao X. Li K. H. Sun Y. J. Zhou W. L. (2019). Characterization of the biosynthetic pathway of nucleotide sugar precursor UDP-glucose during sphingan WL gum production in *Sphingomonas* sp. WG. *J. Biotechnol.* 302 1–9. 10.1016/j.jbiotec.2019.06.005 31199955

[B34] Li H. Li J. Zhou W. Jiao X. Sun Y. Shen Y. (2018). An efficient production of a novel carbohydrate polymer Sphingan WL. *J. Chem. Technol. Biotechnol.* 93 3472–3482. 10.1002/jctb.5705

[B35] Li H. Li K. Guo Z. Xue H. Li J. Ji S. (2021). The Function of beta-1,4-Glucuronosyltransferase WelK in the Sphingan WL Gum Biosynthesis Process in Marine *Sphingomonas* sp. WG. *Mar. Biotechnol.* 23 39–50. 10.1007/s10126-020-09998-9 32979138

[B36] Lyon P. Strippoli V. Fang B. Cimmino L. (2020). B vitamins and one-carbon metabolism: implications in human health and disease. *Nutrients* 12:2867. 10.3390/nu12092867 32961717 PMC7551072

[B37] Maruyama Y. Kobayashi M. Murata K. Hashimoto W. (2015). Formation of a single polar flagellum by two distinct flagellar gene sets in *Sphingomonas* sp. strain A1. *Microbiology* 161 1552–1560. 10.1099/mic.0.000119 26018545

[B38] Mattos-Graner R. O. Duncan M. J. (2017). Two-component signal transduction systems in oral bacteria. *J. Oral Microbiol.* 9:1400858. 10.1080/20002297.2017.1400858 29209465 PMC5706477

[B39] Minamino T. Kinoshita M. Morimoto Y. V. Namba K. (2021). The FlgN chaperone activates the Na(+)-driven engine of the *Salmonella* flagellar protein export apparatus. *Commun. Biol.* 4:335. 10.1038/s42003-021-01865-0 33712678 PMC7955116

[B40] Minnich S. A. Ohta N. Taylor N. Newton A. (1988). Role of the 25-, 27-, and 29-kilodalton flagellins in *Caulobacter crescentus* cell motility: method for construction of deletion and Tn5 insertion mutants by gene replacement. *J. Bacteriol.* 170 3953–3960. 10.1128/jb.170.9.3953-3960.1988 2842293 PMC211395

[B41] Newell P. D. Yoshioka S. Hvorecny K. L. Monds R. D. O’Toole G. A. (2011). Systematic analysis of diguanylate cyclases that promote biofilm formation by *Pseudomonas fluorescens* Pf0-1. *J. Bacteriol.* 193 4685–4698. 10.1128/JB.05483-11 21764921 PMC3165641

[B42] Overhage J. Lewenza S. Marr A. K. Hancock R. E. (2007). Identification of genes involved in swarming motility using a *Pseudomonas aeruginosa* PAO1 mini-Tn5-lux mutant library. *J. Bacteriol.* 189 2164–2169. 10.1128/jb.01623-06 17158671 PMC1855721

[B43] Pelicic V. Reyrat J. M. Gicquel B. (1996). Generation of unmarked directed mutations in mycobacteria, using sucrose counter-selectable suicide vectors. *Mol. Microbiol.* 20 919–925. 10.1111/j.1365-2958.1996.tb02533.x 8809745

[B44] Pini F. De Nisco N. J. Ferri L. Penterman J. Fioravanti A. Brilli M. (2015). Cell cycle control by the master regulator CtrA in *Sinorhizobium meliloti*. *PLoS Genet.* 11:e1005232. 10.1371/journal.pgen.1005232 25978424 PMC4433202

[B45] Rivera-Osorio A. Osorio A. Poggio S. Dreyfus G. Camarena L. (2018). Architecture of divergent flagellar promoters controlled by CtrA in *Rhodobacter sphaeroides*. *BMC Microbiol.* 18:129. 10.1186/s12866-018-1264-y 30305031 PMC6180460

[B46] Sa-Correia I. Fialho A. M. Videira P. Moreira L. M. Marques A. R. Albano H. (2002). Gellan gum biosynthesis in *Sphingomonas paucimobilis* ATCC 31461: genes, enzymes and exopolysaccharide production engineering. *J. Ind. Microbiol. Biotechnol.* 29 170–176. 10.1038/sj.jim.7000266 12355314

[B47] Sánchez-Romero M. A. Casadesús J. (2020). The bacterial epigenome. *Nat. Rev. Microbiol.* 18 7–20. 10.1038/s41579-019-0286-2 31728064

[B48] Schmid J. Ruhmann B. Sieber V. Romero-Jimenez L. Sanjuan J. Perez-Mendoza D. (2018). Screening of c-di-GMP-regulated exopolysaccharides in host interacting bacteria. *Methods Mol. Biol.* 1734 263–275. 10.1007/978-1-4939-7604-1_21 29288461

[B49] Schmid J. Sieber V. Rehm B. (2015). Bacterial exopolysaccharides: biosynthesis pathways and engineering strategies. *Front. Microbiol.* 6:496. 10.3389/fmicb.2015.00496 26074894 PMC4443731

[B50] Schmid J. Sperl N. Sieber V. (2014). A comparison of genes involved in sphingan biosynthesis brought up to date. *Appl. Microbiol. Biotechnol.* 98 7719–7733. 10.1007/s00253-014-5940-z 25081553

[B51] Skerker J. M. Prasol M. S. Perchuk B. S. Biondi E. G. Laub M. T. (2005). Two-component signal transduction pathways regulating growth and cell cycle progression in a bacterium: a system-level analysis. *PLoS Biol.* 3:e334. 10.1371/journal.pbio.0030334 16176121 PMC1233412

[B52] Smith T. G. Hoover T. R. (2009). Deciphering bacterial flagellar gene regulatory networks in the genomic era. *Adv. Appl. Mircobiol.* 67 257–295. 10.1016/S0065-2164(08)01008-3 19245942

[B53] Spangler C. Bohm A. Jenal U. Seifert R. Kaever V. (2010). A liquid chromatography-coupled tandem mass spectrometry method for quantitation of cyclic di-guanosine monophosphate. *J. Microbiol. Methods* 81 226–231. 10.1016/j.mimet.2010.03.020 20385176

[B54] Thorne L. Mikolajczak M. J. Armentrout R. W. Pollock T. J. (2000). Increasing the yield and viscosity of exopolysaccharides secreted by *Sphingomonas* by augmentation of chromosomal genes with multiple copies of cloned biosynthetic genes. *J. Ind. Microbiol. Biotechnol.* 25 49–57. 10.1038/sj.jim.7000019

[B55] Tsang J. Hoover T. R. (2014). Themes and variations: regulation of RpoN-dependent flagellar genes across diverse bacterial species. *Scientifica* 2014:681754. 10.1155/2014/681754 24672734 PMC3930126

[B56] Vega-Baray B. Domenzain C. Rivera A. Alfaro-López R. Gómez-César E. Poggio S. (2015). The flagellar set Fla2 in *Rhodobacter sphaeroides* is controlled by the CckA pathway and is repressed by organic acids and the expression of Fla1. *J. Bacteriol.* 197 833–847. 10.1128/jb.02429-14 25512309 PMC4325097

[B57] Wang S. W. Yu S. Zhang Z. Y. Wei Q. Yan L. Ai G. M. (2014). Coordination of swarming motility, biosurfactant synthesis, and biofilm matrix exopolysaccharide production in *Pseudomonas aeruginosa*. *Appl. Environ. Microb.* 80 6724–6732. 10.1128/Aem.01237-14 25172852 PMC4249032

[B58] Wilkins M. R. Gasteiger E. Bairoch A. Sanchez J. C. Williams K. L. Appel R. D. (1999). Protein identification and analysis tools in the ExPASy server. *Methods Mol. Biol.* 112 531–552. 10.1385/1-59259-584-7:531 10027275

[B59] Willett J. W. Herrou J. Briegel A. Rotskoff G. Crosson S. (2015). Structural asymmetry in a conserved signaling system that regulates division, replication, and virulence of an intracellular pathogen. *Proc. Natl. Acad. Sci. U. S. A.* 112 E3709–E3718. 10.1073/pnas.1503118112 26124143 PMC4507200

[B60] Willis L. M. Whitfield C. (2013). Structure, biosynthesis, and function of bacterial capsular polysaccharides synthesized by ABC transporter-dependent pathways. *Carbohyd. Res.* 378 35–44. 10.1016/j.carres.2013.05.007 23746650

[B61] Xu X. P. Nie Z. M. Zheng Z. Y. Zhu L. Zhang H. T. Zhan X. B. (2017). Different nitrogen sources change the transcriptome of welan gum-producing strain *Sphingomonas* sp ATCC 31555. *Arch. Microbiol.* 199 1055–1064. 10.1007/s00203-017-1372-3 28396915

[B62] Yamazaki M. Thorne L. Mikolajczak M. Armentrout R. W. Pollock T. J. (1996). Linkage of genes essential for synthesis of a polysaccharide capsule in *Sphingomonas* strain S88. *J. Bacteriol.* 178 2676–2687. 10.1128/jb.178.9.2676-2687.1996 8626338 PMC177995

[B63] Zhou B. Schrader J. M. Kalogeraki V. S. Abeliuk E. Dinh C. B. Pham J. Q. (2015). The global regulatory architecture of transcription during the *Caulobacter* cell cycle. *PLoS Genet.* 11:e1004831. 10.1371/journal.pgen.1004831 25569173 PMC4287350

[B64] Zhou W. L. Jiao X. Sun Y. J. Li H. Zhu H. (2017). Determination of extracellular polysaccharides produced by marine derived *Sphingomonas* sp. WG. *Chin. J. Mar. Drugs* 36 27–34.

