# Supplementary material for: Hybrid Histidine Kinase WelA of Sphingomonas sp. WG Contributes to WL Gum Biosynthesis and Motility
Source: Front Microbiol. 2022 Mar 1;13:792315. doi: 10.3389/fmicb.2022.792315 (PMC8921679; doi:10.3389/fmicb.2022.792315)
Supplement: Supplementary file 1 [file Table_1.docx]

Supplementary Material

**Hybrid** **Histidine Kinase WelA of *Sphingomonas* sp. WG to WL Gum Biosynthesis and Motility**

**Hui Li^1^, Mengqi Chen^1^, Zaimei Zhang^1^, Benchao Li^1^, Jianlin Liu^1^, Han Xue^1^, Sixue Ji^1^, Zhongrui Guo^1^, Jiqian Wang ^1^* and Hu Zhu ^1, 2^***

^1^ State Key Laboratory of Heavy Oil Processing and Centre for Bioengineering and Biotechnology, China University of Petroleum (East China), 66 Changjiang West Road, Qingdao 266580, People’s Republic of China

^2^ Engineering Research Center of Industrial Biocatalysis, Fujian Province Universities, College of Chemistry and Materials Science, Fujian Normal University, 32 Shangsan Road, Fuzhou 350007, People’s Republic of China

*** For Correspondence:**

Jiqian Wang: jqwang@upc.edu.cn; Hu Zhu: zhuhu@fjnu.edu.cn. Tel.: +86-532-86981563

**
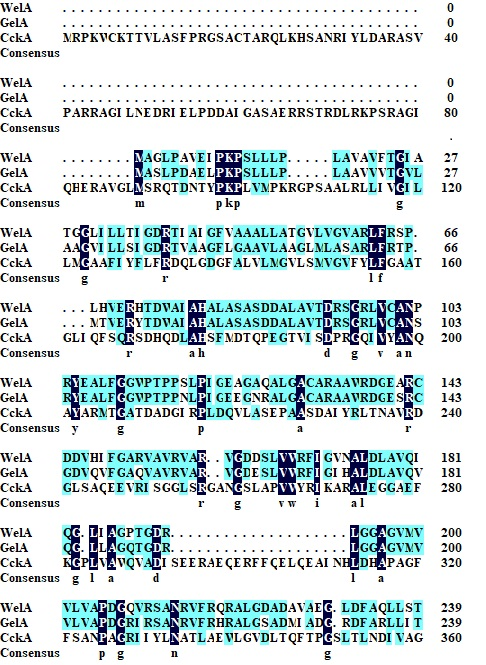
**

**
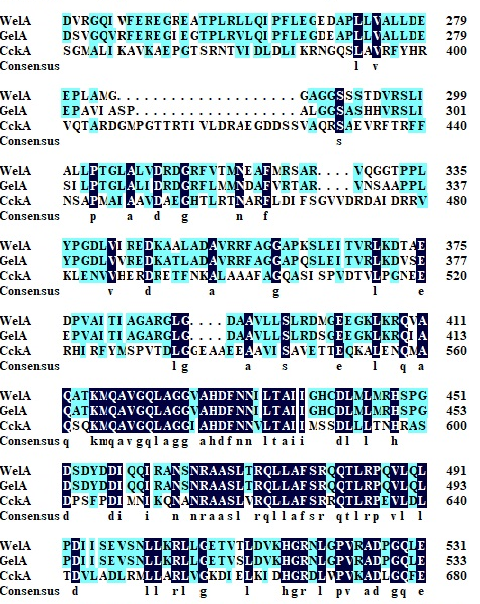
**

**
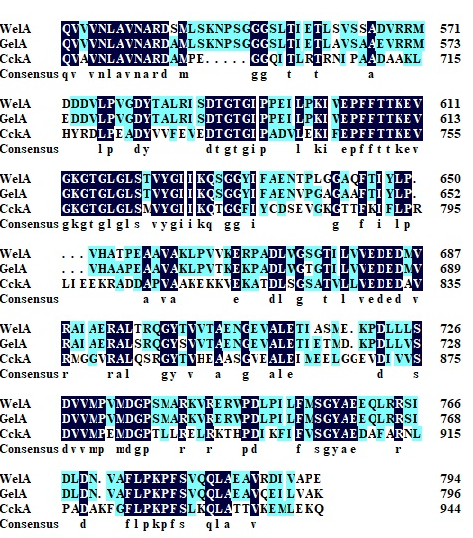
**

Figure 1 Comparison of amino acid sequences of WelA, GelA (WP 010545582) and CckA (P0DOA0.1)


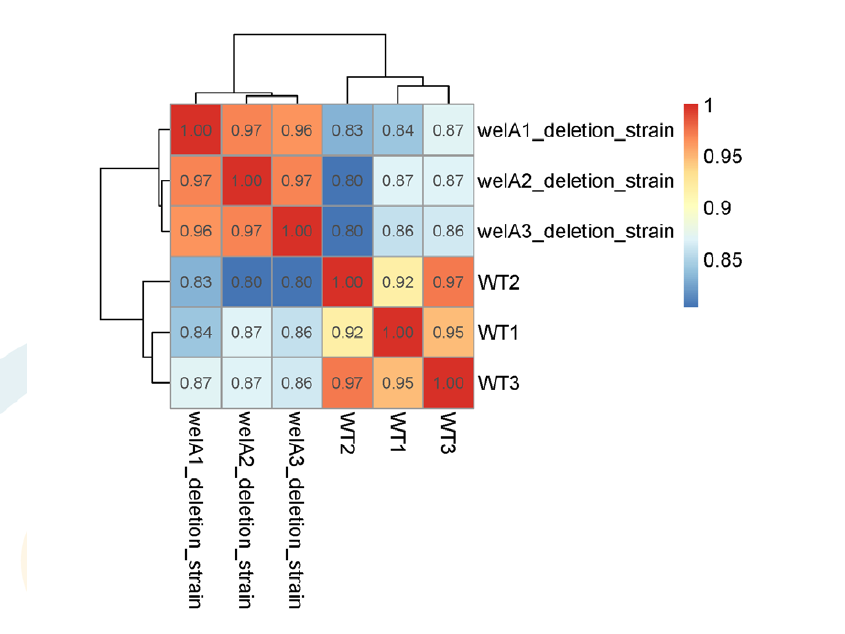


Figure 2 The heat map of correlation chart between samples


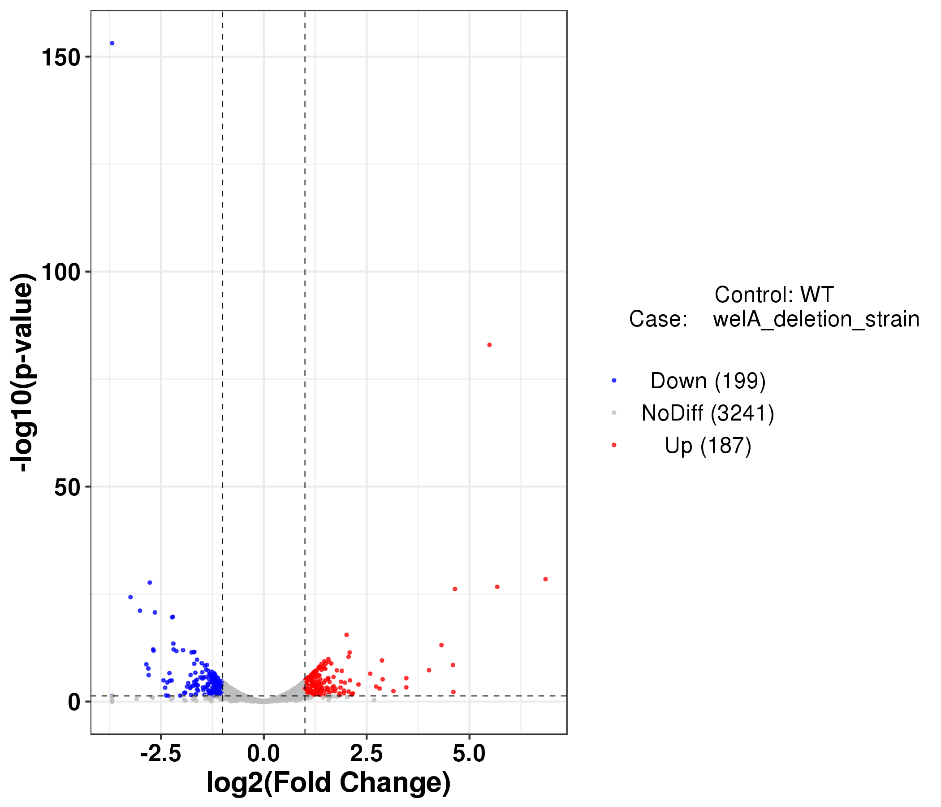


Figure 3 The volcano map of DEGs analyzed in WT and *welA* deletion strain


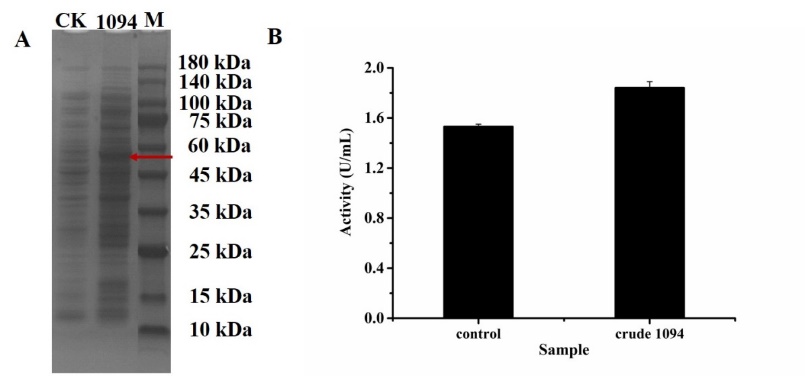


Figure 4. Heterologous expression and enzyme activity of ORF1094. (A) SDS-PAGE analysis of ORF1094 encoding protein fused with a Trx and 6×His tag with an expected molecular weight of 47 kDa. (B) Enzyme activity of ORF1094





Figure 5. The intracellular concentration of c-di-GMP in WT and ∆*welA* mutant strain

Table1 Primers used in the construction of deletion and complemented strains

| Primers | Sequence (5’-3’) | |
| --- | --- | --- |
| welA5flFor | AGGTGCCAGAAATTGCCGCGGACTT |  |
| welA5flRev | GCACATCGGTGGCGGGTGCTTGGCATGCAACAGTTTTC | |
| welA3flFor | AACTGTTGCATGCCAAGCACCCGCCACCGATGTGCCGTTGT | |
| welA3flRev | AAGCGCGCAGTCGATTTCACCACC | |
| welAdelFor | AATGAGCTCCGCCCAGTCCATCGAACAG | |
| welAdelRev | GCCTCTAGACTCGTGAAGAACGTGATCGCC | |
| welAinFor | CGAGTGCCGCATTAGCATCAGGT | |
| welAinRev | CTGCGGCTCCTTCAGATTCCGTTTC | |
| welAexFor | AAACTCGAGGATGGCCGGTCTGCCAGCTGT | |
| welAexRev | GCTCTAGATTATTCTGGCGCTACGATGTCC | |
| Gene1094exFor | CGCGGATCCATGGTTCTCGATCAGGAGGACTT | |
| Gene1094exRev | CCCAAGCTTTCATGCGTTGGCGGTGGGAC | |

Table 2 Primers used in the real-time PCR

| Primers | Sequence (5'to 3') |
| --- | --- |
| Gene1768qrtPCRFor | CTCAAGGGCTATGGCGTGCT |
| Gene1768qrtPCRRev | CATTGGCGGTTCTCGTCG |
| Gene2565qrtPCRFor | CGATGTCGTGCTGGAGATGA |
| Gene2565qrtPCRRev | CGCCTTTGGCAATGGTGA |
| Gene1094qrtPCRFor | AGGACGGCGACGAGGTTTTC |
| Gene1094qrtPCRRev | CGGCGGTCATTGCCAGATAG |
| Gene3600qrtPCRFor | AGGCATTTCCCTATTCCCGC |
| Gene3600qrtPCRRev | GCCCTTCCTTCATCACCACG |
| Gene3601qrtPCRFor | CGGTATCGCTGTGGGAAGT |
| Gene3601qrtPCRRev | CAGGGCAAGCAGATGGTG |
| Gene958qrtPCRFor | GGTGGGGATGAGCGAGA |
| Gene958qrtPCRRev | GCCTCAATGGTGGCGTTA |
| Gene1411qrtPCRFor | GACGAACTGGTTGCCCG |
| Gene1411qrtPCRRev | CCTTGCGAAGCGAGAGC |
| Gene1294qrtPCRFor | AGAGAGAGCGACTTCTTGCG |
| Gene1294qrtPCRRev | CCCTCATACGAGCCCACAT |
| Gene3486qrtPCRFor | GACATGGTGCAGAAGGTGGAT |
| Gene3486qrtPCRRev | CAAGTTGGACAGAAGGGCGT |
| Gene592qrtPCRFor | GTTGATGCCGACCACAGG |
| Gene592qrtPCRRev | TCCGTTCGCCCCAGATAC |
| 16SqPCRFor^1^ | CAGCGTTTGACATGGTAGGAC |
| 16SqPCRRev | TAACCCAACATCTCACGACAC |

^1^ the Primers for 16S rRNA was the same in our previous work^1^

**Table 3 The data of the sequencing**

| **Sample** | **Reads Num.** | **Bases (bp)** | **Clean Reads No.** | **Clean Data (bp)** | **Clean Reads (%)** | **Clean Data (%)** | **Q20 (%)** | **Q30 (%)** |
| --- | --- | --- | --- | --- | --- | --- | --- | --- |
| **∆***welA*1 | 39967356 | 5995103400 | 37456122 | 5618418300 | 93.71 | 93.71 | 96.96 | 92.16 |
| **∆***welA*2 | 39967708 | 5995156200 | 37012532 | 5551879800 | 92.60 | 92.60 | 96.96 | 92.23 |
| **∆***welA*3 | 36478476 | 5471771400 | 34351600 | 5152740000 | 94.16 | 94.16 | 97.19 | 92.64 |
| WT1 | 40321632 | 6048244800 | 34348492 | 5152273800 | 85.18 | 85.18 | 97.90 | 94.58 |
| WT2 | 30398256 | 4559738400 | 28183986 | 4227597900 | 92.71 | 92.71 | 96.92 | 92.12 |
| WT3 | 38205176 | 5730776400 | 34699050 | 5204857500 | 90.82 | 90.82 | 96.92 | 92.11 |

Table 4 The expressional level of DEGs involved in flagellar assembly and motility

| Gene ID | Gene product description | Log_2_FC | *p*-value |
| --- | --- | --- | --- |
| Gene 249 | Flagellar hook protein FlgE | 1.86 | 0.00053 |
| Gene250 | flagellar hook-associated protein FlgK | 1.52 | 0.034 |
| Gene251 | flagellin | 1.29 | 0.0089 |
| Gene264 | flagellar basal body rod protein FlgF | 1.53 | 0.00059 |
| Gene265 | Flagellar basal-body rod protein FlgG | 1.42 | 0.00060 |
| Gene266 | flagellar basal body P-ring biosynthesis protein FlgA | 1.73 | 0.0038 |
| Gene267 | flagellar L-ring protein FlgH | 1.06 | 0.0027 |
| Gene270 | Basal-body rod modification protein FlgD | 1.56 | 0.0010 |
| Gene271 | flagellar hook-length control protein FliK | 1.21 | 0.016 |
| Gene272 | flagellar biosynthesis protein FlhA | 1.40 | 0.024 |
| Gene273 | Transcriptional regulatory protein FlbD | 1.55 | 0.0017 |
| Gene274 | Flagellar motor switch protein FliN | 2.17 | 0.013 |
| Gene 275 | flagellar M-ring protein FliF | 1.35 | 0.024 |
| Gene280 | Regulatory protein FlaEY | 1.20 | 0.0070 |
| Gene282 | Hypothetical protein | -1.38 | 3.09E-09 |
| Gene283 | flagellar protein FlgN | 1.09 | 0.0053 |
| Gene285 | flagellar basal body P-ring protein FlgI | 1.16 | 0.0061 |
| Gene586 | Flagellar hook-basal body complex protein FliE | -1.26 | 0.0043 |
| Gene2514 | flagellar regulatory FlaF family protein | 1.21 | 0.0062 |
| Gene958 | methyl-accepting chemotaxis sensory transducer | 2.59 | 3.3E-07 |
| Gene959 | twitching motility protein PilJ | 2.87 | 2.7E-10 |
| Gene697 | twitching motility protein PilT | -2.34 | 3.5E-05 |
| Gene3139 | type III secretion system ATPase FliI | -2.86 | 2.1E-09 |
| Gene3258 | type II toxin-antitoxin system VapC family toxin | -1.18 | 0.00076 |
| Gene3486 | Chemoreceptor McpA | -1.54 | 2.2E-06 |
| Gene3280 | chemotaxis protein CheR | -1.02 | 0.0023 |
| Gene2292 | Flp/Fap pilin component | 1.40 | 7.4E-05 |

Reference

1. Li, H.; Li, J.; Zhou, W.; Jiao, X.; Sun, Y.; Shen, Y.; Qian, J.; Wang, J.; Zhu, H., An efficient production of a novel carbohydrate polymer Sphingan WL. *J Chem Technol Biotechnol* **2018,** *93* (12), 3472-3482.
